# The timing of goniosynechialysis in treatment of primary angle-closure glaucoma combined with cataract

**Published:** 2012-04-27

**Authors:** Jun Yu, Min Sun, Yanli Wei, Xiaofeng Cai, Chunyan He, Xiaoju An, Jian Ye

**Affiliations:** Department of Ophthalmology, Research Institute of Surgery & Daping Hospital, Third Military Medical University, Chongqing, P.R. China

## Abstract

**Purpose:**

To compare the clinical effects of phacoemulsification (PHACO) combined with goniosynechialysis (GSL) at different times in the treatment of primary angle-closure glaucoma (PACG) combined with cataract.

**Methods:**

Before surgery, one or more different kinds of anti-glaucoma medicines were used for 24 patients (32 eyes) of PACG combined with cataract. A combination of PHACO with GSL procedures were performed on both groups of patients. The patients were randomly divided into two groups: 17 patients with 21 eyes were in Group A (GSL performed before lens was removed) and 7 patients with 11 eyes in Group B (GSL after extraction of crystal cortex). Changes in visual acuity, intraocular pressure (IOP) and the depth of the center anterior chamber were observed before surgery and again at 1 month, 3 months, 6 months, and 12 months after surgery.

**Results:**

The mean visual acuity of Group A and Group B was 1.13±0.75 and 0.93±0.50, respectively. There was no statistical difference between these two groups in visual acuity before surgery. At 1 month, 3 months, 6 months, and 12 months after surgery, the visual acuities in Group A were 0.57±0.33, 0.42±0.24, 0.30±0.23, 0.35±0.28 and the visual acuities in Group B were 0.68±0.60, 0.38±0.15, 0.40±0.17,0.33±0.13, and 0.37±0.06. Visual acuity after surgery was greatly improved in both groups. However, there was no difference between these two groups at the different points in time mentioned above. The mean IOP before surgery was 35.67±12.31 mmHg and 31.64±15.06 mmHg for Group A and Group B, respectively. At 1 month, 3 months, 6 months, and 12 months after surgery, the IOP were normalized and were significantly lower than before surgery, in group A and B. However, there was no difference in IOP between these groups at the different points in time as mentioned above. One year after surgery, the percentages of success in Group A and Group B were 86.0% and 90.0%, respectively, qualifid success rates in Group A and Group B were 9.5% and 10.0%, respectively. The failure rate in Group A was 4.8%, and no one failed in Group B. In Group A, the number of medications pre-operation was 2.05±0.74. A trabeculectomy was performed on 1 eye, and anti-glaucoma medicines were used for 2 eyes after surgery to normalize IOP. In Group B, the number of medications pre-operation was 2.18±0.87. One anti-glaucoma medicine- was used for 1 eyes. In different period after surgery, anterior chamber angles in Group A were all open. Narrow anterior chamber angles in different extents also were observed in 4 eyes in Group B. The mean depth of the center anterior chamber before surgery was 1.56±0.37 mmHg and 1.72±0.35 mmHg for Group A and Group B, respectively. At 1 month, 3 months, 6 months and 12 months after surgery, the center anterior chamber was deeper than that before surgery both in both groups . However, there was no difference in the center anterior chamber’s depth between these groups at the different points in time mentioned above.

**Conclusions:**

For PACG patients with cataracts, surgery methods are shown to improve visual acuity, decrease IOP, and expand the anterior chamber angle. Regarding the opening extent of the anterior chamber angle, surgery performed on Group A achieved better results than Group B.

## Introduction

As previously documented, long-standing peripheral anterior synechiae (PAS) were associated with permanent trabecular damage [1,2]. It was reported that goniosynechialysis (GSL) was performed by Shaffer [3] to treat angle-closure glaucoma in 1957. Thereafter, the surgery has been continually improved. In 1984, the method proposed by Campbell, et al. [1] to expand the chamber angle using viscoelastic material in surgery was in practice. In the Campbell et al. [1,4] method, GSL is performed after the lens is removed. Additionally, a viscoelastic agent was used to expand the chamber angle. The surgical results varied according to the reports. In our study of the timing of GSL in the surgery, we performed GSLs before and after PHACOs to compare the effects of two types of surgeries so as to find the best way to treat primary angle-closure glaucoma (PACG) combined with cataract.

## Methods

### General information

**Figure 1:**
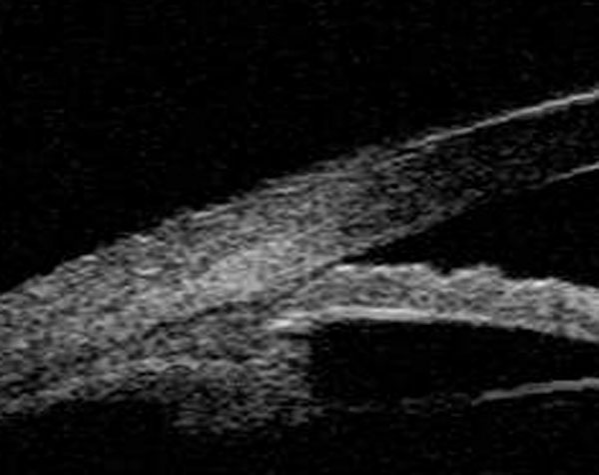
The group A show the chamber angle are closed pre-operation (in terms of UBM).

**Figure 2:**
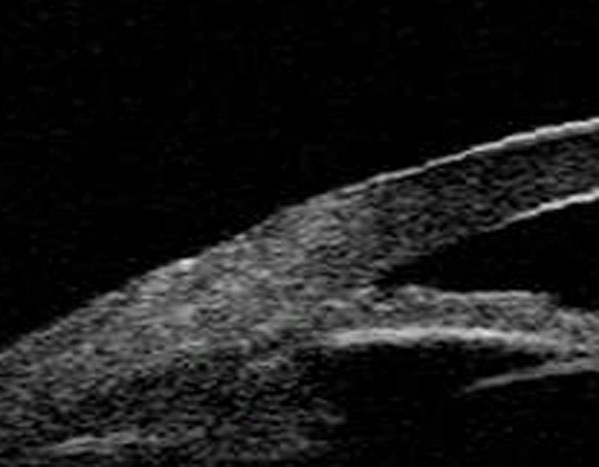
The group B show the chamber angle are closed pre-operation (in terms of UBM).

This study adhered to the tenets of the Declaration of Helsinki. All participants signed the respective informed consent forms. The research was approved by the Ethics Committee of the Research Institute of Field Surgery, Da Ping Hospital, Third Military Medical University, Chongqing, P.R. China. Thirty-nine patients (52 eyes) with chronic angle-closure glaucoma with cataract were admitted to participate and were treated by us from June 2010 to June 2011: all patients were randomized into 2 groups (Group A or Group B) using a random number table created by SPSS statistical software (SPSS version 13.0; SPSS, Chicago, IL). Group A consisted of 20 patients and Group B consisted of 19 patients. Eight patients in Group A had PACG and cataracts in both eyes, and 5 patients in Group B had PACG and cataracts. Fifteen patients did not return to us during the study. Among the remaining 24 participants, 4 patients from both Group A and Group B suffered PACG and cataracts in both eyes Ten patients (14 eyes) were male and 14 cases (18 eyes) were female. The mean age of participants was 67.4±5.6 years. The lenses of all patients were nepheloid to different degrees. The degrees of cataract in all patients’ eyes were CII or CIII [5] according to the Lens Opacities Classification System, version II (LOCS II). The degrees of nuclear hardness in all eyes were equal to or more than grade III according to the Emery and Little nuclear hardness classification. According to the results of gonioscope and ultrasound biomicroscopy (UBM) examinations before surgery, anterior chamber angles had narrowed to different degrees (Figure 1 and Figure 2). The visual acuities of 10 eyes were between light perception and 0.1. The visual acuities of the other 24 eyes were between 0.1 and 0.5. The comparison of gender, age and visual acuity of patients between these two groups was free of statistical difference.

### Case series

#### The inclusion criteria were as follows

1. Occludable angle confirmed by gonioscopy (OMVGLF-1.5X): the gonioscope examinations were performed in a darkened room with minimum-possible slit-lamp illumination, making sure that the slit beam did not impinge upon the pupil [6]. The extent of posterior goniosynechia of the included patient was one-third to three-quarters.

2. Evidence was found of uncontrolled IOP (raised IOP, progressive glaucomatous optic neuropathy, and/or visual field progression).

3. All patients conformed to the diagnose standard of angle-closure glaucoma proposed by Foster et al. [7] in 2002: people meeting gonioscopic criteria for narrow angles and with evidence of significant obstruction of the functional trabecular meshwork by the peripheral iris would be classified as having primary angle closure (PAC). Those in whom PAC had led to significant glaucomatous damage to the optic nerve would be defined as having PACG.

#### Exclusion criteria included

1. Plateau iris syndrome.

2. Previous glaucoma surgery (argon laser/trabeculoplasty/trabeculectomy).

3. Retinal disease.

4. History of ocular injury.

5. Previous patent PI.

6. Other glaucomas.

Preoperatively and postoperatively, all patients underwent complete ocular examination.

#### Treatment

Phacomulsification, intraocular lens implantation, and GSL were performed on 39 patients. Phacomulsification was performed as the usual method of surgical operation, but 1 ml viscoelastic material was used, rather than 0.5 ml. In Group A, after an incision was performed on the transparent cornea, viscoelastic material was filled in the anterior chamber to achieve more depth at the anterior chamber. Next approximately 0.5 ml viscoelastic material was placed near the anterior angle to blunt dissect the anterior chamber in 360° and separate PAS. In Group B, after an incision was performed on the transparent cornea, a small amount of viscoelastic material was filled as described in the usual practice to achieve more depth at the anterior chamber. After the removal of lens cortical material by an irrigation-aspiration (I/A) probe, about 0.5 ml viscoelastic material was placed near the anterior angle to blunt dissect the anterior chamber in 360° and separate PAS. In both Group A and Group B, it was ensured that the tip did not have contact with trabecular meshwork. In the two types of surgeries, gonioscope (O4MAC) was used to observe the opening degree of the anterior angle after GSL. If any residual PAS was found at the root of the iris after GSL, a blunt instrument was used to press the root of the iris and resolve the remaining PAS. Eye patches of all patients were removed one day after the surgery. Hormone eye drops and non-corticosteroid eye drops were prescribed for the eyes.

#### Visits

Visual acuity, IOP, slit lamp, UBM, and a rotating Scheimpflug camera (Pentacam) were performed before surgery and at 1 month, 3 months, 6 months and 12 months after the surgery. The technicians who performed the IOP examination, Pentacam, UBM et al., were masked to group assignment.

### Statistical handling

Data were expressed as the mean ±standard deviation (SD). A p value below 0.05 was taken as a statistically significant difference. The analysis was performed using SPSS software (SPSS version 13.0; SPSS, Chicago, IL).

## Results

### Visual acuity

**Figure 3:**
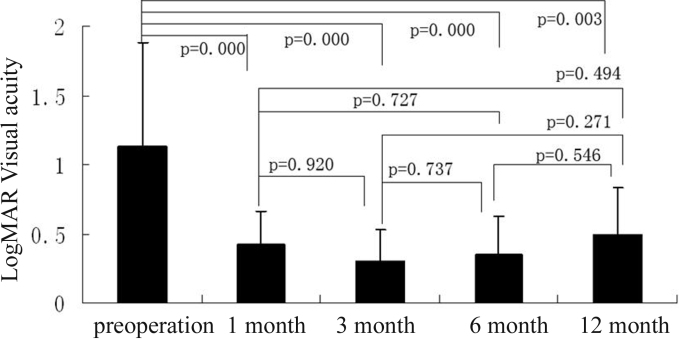
In group A, the best-corrected visual acuity (in terms of logMAR) pre-operation and at 1 month, 3 month, 6 month, and 12 month after operation, the bars mean the average of the test, the error bars mean standard deviation.

**Figure 4:**
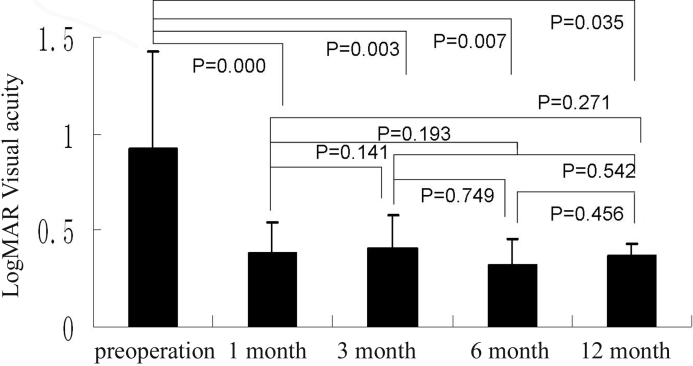
In group B, the best-corrected visual acuity (in terms of logMAR) pre-operation and at 1 month, 3 month, 6 month, and 12 month after operation, the bars mean the average of the test, the error bars mean standard deviation.

The mean visual acuities of Group A and Group B were 1.13±0.75 and 0.93±0.50 respectively. There was no statistical difference between these two groups in visual acuity before surgery. At 1 month, 3 months, 6 months, and 12 months after surgery, the visual acuities in Group A were 0.57±0.33 (p=0.000), 0.42±0.24 (p=0.000), 0.30±0.23 (p=0.000), 0.35±0.28 (p=0.003; Figure 3). The visual acuities in Group B were 0.38±0.15 (p=0.000), 0.40±0.17 (p=0.003), 0.33±0.13 (p=0.007), 0.37±0.06 (p=0.035; Figure 4). Visual acuity of all patients in both groups increased after the surgery. No appreciable difference (p>0.05) was observed in the visual acuity between these two groups at 1 month, 3 months, 6 months, and 12 months after the surgery.

### IOP and anti-glaucoma medicine for decreasing IOP

**Figure 5:**
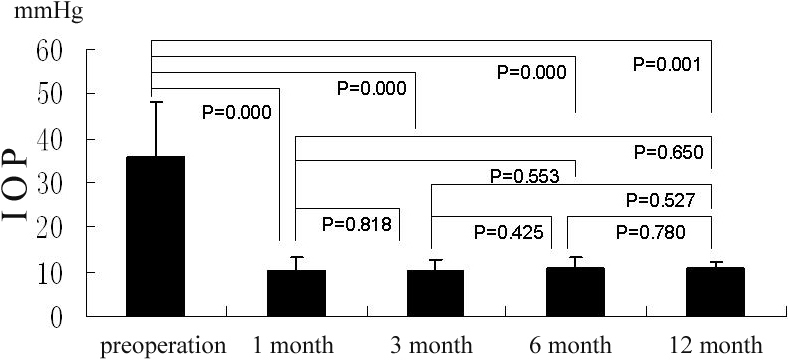
In group A, the IOP (in terms of Goldman IOP) pre-operation and at 1 month, 3 month, 6 month, and 12 month after operation, the bars mean the average of the test, the error bars mean standard deviation.

**Figure 6:**
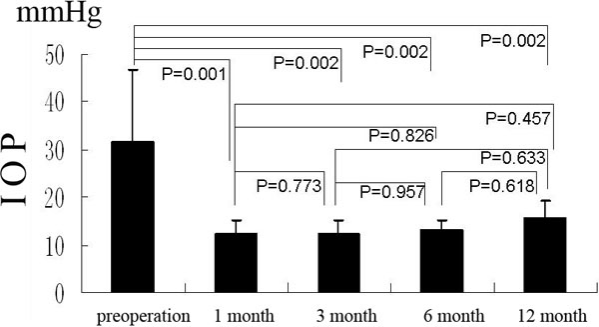
In group B, the IOP (in terms of Goldman IOP) pre-operation and at 1 month, 3 month, 6 month, and 12 month after operation, the bars mean the average of the test, the error bars mean standard deviation.

Before surgery, the mean IOP was 35.67±12.31 mmHg and 31.64±15.06 mmHg for groups A and B, respectively. At 1 month, 3 months, and 6 months after surgery, the IOPs of Group A were 10.33±2.80 (p=0.000), 10.13±2.58 (p=0.000), 11.00±2.27 (p=0.000), 10.00±1.41 (p=0.001; Figure 5). The IOPs of Group B were 12.20±2.90 (p=0.001), 12.13±2.95 (p=0.002), 13.00±2.31 (p=0.002), and 15.67±3.79 (p=0.002; Figure 6). The IOPs of all patients were normalized and significantly lower than before surgery both in Group A and Group B. However, there was no difference of IOP between these groups at the different time points mentioned above.

In our study, complete success was defined as IOP remaining <21 mmHg with no additional medication, while qualified success was defined as those requiring additional anti-glaucoma medication, and failure was defined as

1. Patients’ postoperative visual acuity dropping to no light perception,
2. IOP> 21 mmHg in two consecutive follow-ups on patients with anti-glaucoma medications,
3. and patients who required another anti-glaucoma operation [8].

In Group A, one eye showed increased IOP again on the third day after surgery. Anti-glaucoma medicine was prescribed to decrease the IOP but had no effect. A trabeculectomy was performed on the eye. IOP was controlled within the normal scope after the surgery. IOP of one eye fluctuated between 20 mmHg and 25 mmHg after the surgery. Two kinds of anti-glaucoma medicines were prescribed as treatment and the IOP was normalized. The IOP of one eye was 21 mmHg at 11 months after the surgery. Anti-glaucoma medicine was applied locally as treatment and the IOP was controlled within the normal scope. No further anti-glaucoma medicines were used on the other patients.

In Group B, 2 eyes showed bleeding during surgery when blunt instruments were used to separate the root of the iris. When hemostasis therapy was taken after the surgery, hematocele in the anterior chamber was completely absorbed. Three eyes showed raised IOP on the first day after the surgery, fluctuating between 21 mmHg and 31 mmHg. One patient’s eye’s IOP needed anti-glaucoma medicine to control IOP and the patient’s eyes IOP became normal on the second day after surgery.

### UBM observation

After surgery, iris roots of 21 eyes in Group A opened up more or less. In Group B, 7 out of 11 eyes (including separation due to mechanical pressing for 4 eyes) opened up, and the other 4 eyes developed synechia after surgery.

### The depth of the center anterior chamber

**Figure 7:**
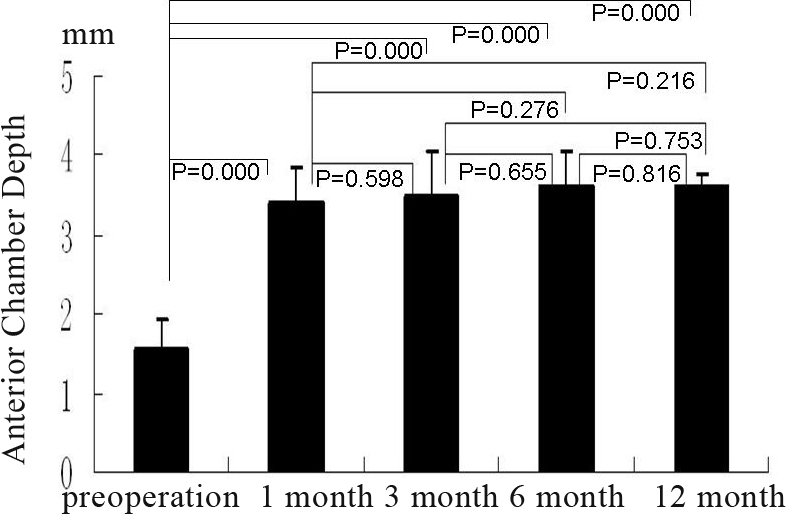
The group A shows the anterior chamber depth (in terms of Pentacam) pre-operation and at 1 month, 3 month, 6 month, and 12 month after operation, the bars mean the average of the test, the error bars mean standard deviation.

**Figure 8:**
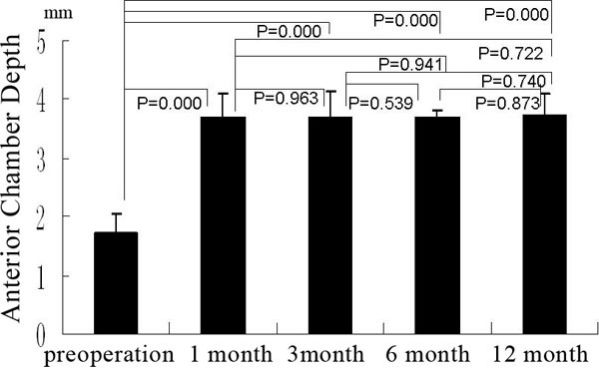
The group B shows the anterior chamber depth (in terms of Pentacam) pre-operation and at 1 month, 3 month, 6 month, and 12 month after operation, the bars mean the average of the test, the error bars mean standard deviation.

The mean depth of the center anterior chamber before surgery was 1.56±0.37 mmHg and 1.72±0.35 mmHg for Group A and Group B, respectively. At 1 month, 3 months, 6 months, and 12 months after surgery, the depths of Group A were 3.40±0.46 (p=0.000), 3.50±0.57 (p=0.000), 3.59±0.48 (p=0.000), 3.59±0.19 (p=0.000; Figure 7), and the depth of Group B were 3.69±0.42 (p=0.000), 3.69±0.45 (p=0.000), 3.67±0.16 (p=0.000), and 3.75±0.35 (p=0.000; Figure 8). The depth of all patients’ center anterior chamber was deeper than that before surgery in both Group A and Group B. However, there was no difference in the center anterior chamber’s depth between these groups at different points in time, as mentioned above.

### The change of the visual field

In this study, we used the mean deviation (MD, in db; measured with the Humphrey Field Analyzer) to evaluate the visual field abnormality. MD values reflected extensive damage of the retinas. The more severe the damage to the retina was, the lower the value of the MD. Each p value corresponds to a MD value. The p value indicates the probability of that MD being found in the normal population. The MD in Group A ranged from 3.68 db to 24.13 db, with a mean value of 10.55 db (SD=7.14) ; in Group B the MD ranged from 3.82 db to 24.92 db, with a mean value of 11.93 db (SD=6.08) before surgery. At 1 month, 3 months, 6 months, and 12 months after surgery, the MD values were lower than that before surgery. However, there was no statistical significance in MD and corresponding p value between different time points.

### The change of the cup/disc ratio

In this study, we used the cup/disc ratio (C/D; measured with the ZEISS Stratus OCT 3000; Carl Zeiss Meditec, Dublin, CA) to evaluate the C/D ratio. The more severe the optic nerve damage, the higher the value of C/D. The C/D in Group A ranged from 0.38 db to 0.87, with a mean value of 0.61 (SD=0.16); in Group B the C/D ranged from 0.45 db to 0.78, with a mean value of 0.65 (SD=0.11) before surgery. At 1 month, 3 months, 6 months, and 12 months after surgery, the C/D value were almost the same as before surgery in both groups A and B, with no statistical significances.

### Complications

Corneal edema developed in the patients of both groups after surgery, with a 100% ratio of occurrence. No bleeding occurred in the anterior chamber for Group A during the surgery. The iris roots of 4 eyes in Group B were mechanically pressed during in the surgery to separate PAS, and 2 eyes showed a small amount of bleeding in the anterior chamber on the second day after surgery. The blood was completely absorbed on the third day after the surgery.

## Discussion

**Figure 9:**
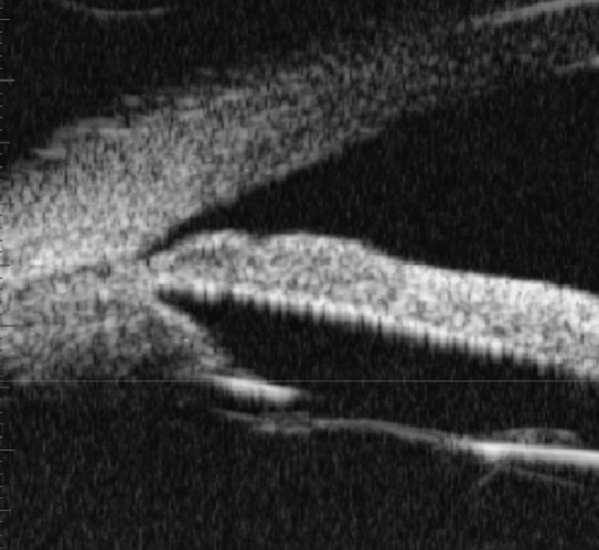
The group B show the chamber angle are opening at 12 month after operation (in terms of UBM).

**Figure 10:**
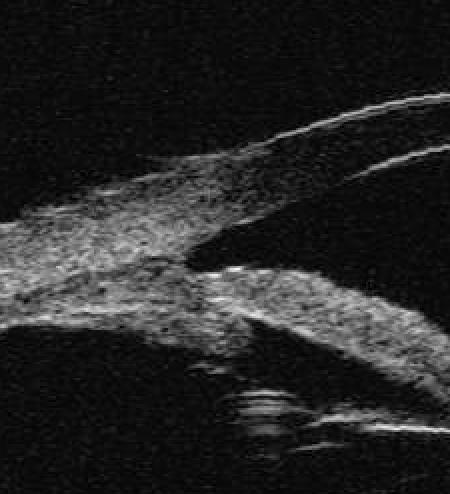
The group A show the chamber angle are opening at 12 month after operation (in terms of UBM).

Clinically, more and more doctors begin to accept PHACO combined with GSL as the usual surgery to treat PACG with cataract [9,10]. The general outcome of the surgery was positive with few side effects. Most patients no longer needed to use anti-glaucoma medicines, which could in turn result in less financial stress for them. However, there still remains the question of when to perform this surgery to achieve the best results. According to the documentations [2,4,9-11], all the doctors choose to perform GSL after phacoemulsification, and the rate of success varied [12-14]. Through clinical observation during the surgery, we find that the central anterior chamber in Group B was significantly deeper than in Group A during the surgery. This may be the reason why so many surgeons prefer to perform GSL after the crystalline lens is removed. After surgery, the results of UBM and Pentacam examinations showed that the central anterior chambers of patients in Group B are truly deeper than those in Group A, but the opening degree of the chamber angle (Figure 9) is poorer than those in Group A (Figure 10). In Group B, the iris roots of some patients are not sufficiently separated. The reasons for this might be that when GSL is performed on patients in Group B, the lens is not available, as the supporting points and the motion space at the pupillary margin for the iris is quite large. When a certain amount of viscoelastic material was filled in, the iris went down and the force acted on the chamber angle is reduced. Some researchers [4] reported that when GSL was performed after the crystalline lens was removed, synechia was appreciably smaller than before surgery according to gonioscope examinations during surgery. Mechanically pressing at the top of the iris root to separate PAS, synechiae at the root of the iris was found through an examination with a gonioscope, which may impair the root of the chamber angle or the iris root and cause certain impairment to the trabecular meshwork. According to the report, the ratio of bleeding in angle separation, caused by pressing the iris root with a blunt instrument, is about 11% [12]. We find that the opening degree of a chamber angle was preferable when the iris root was pressed with instruments in Group B. Additionally, when the complications mentioned above occurred easily, the surgical operation was more difficult to perform. The opening degree of the chamber angle gradually decreased with time in group B. At 12 months after the surgery, the examinations of 4 eyes in 3 patients showed that the opening degrees were significantly smaller than those at 1 month after surgery. The IOP of 2 eyes increased to 20 mmHg. The results of UBM and Pentacam examinations showed that that opening degree of the anterior chamber angle at 12 months after surgery was significantly smaller than at one month post-surgery. As the patients did not return for regular visit, as required, we were not sure whether the close-up of the chamber angle was caused by the anterior chamber's inflammation, the chamber’s bleeding after the surgery, or by the surgery itself [15].

No one has reported the operation method of Group A until now. We guess that the capacity of the anterior chamber is small due to the unremoved expanded crystalline lens, which may result in the limited opening degree of the chamber angle. Moreover, the equal amount of viscoelastic material filling the anterior chamber before the lens was removed may easily increase the load of the lens’s suspensory ligament, resulting in a tear. However, because of the limited downward scope of the peripheral iris as the lens was not removed, the dosage of viscoelastic material placed at the root of chamber angle is more sufficient under the effect of equal dosage of viscoelastic material. When viscoelastic material is filled, it will press both the iris root and the center down, so that the angle can be more sufficiently separated. Meanwhile, tears, bleeding at the iris root, damage to trabecular meshwork and other complications [12] can be reduced.

Others have reported that the effect of GSL was negative .The reasons for the negative effect may be that the separation was not abundant, and the synechia was still existed after surgery. Through our observations, the opening degree of the chamber angle was better in Group A than in Group B. Therefore, we infer that GSL performed before the lens was removed maybe the optional choice for patients of PACG complicated with cataract.

## References

[1] Campbell DG, Vela A (1984). Modern goniosynechialysis for the treatment of synechial angle-closure glaucoma.. Ophthalmology.

[2] Tarongoy P, Ho CL, Walton DS (2009). Angle-closure Glaucoma:The Role of the Lens in the Pathogenesis,Prevention,and Treatment.. Surv Ophthalmol.

[3] Shaffer RN (1957-1958). Operating room gonioscopy in angle closure glaucoma surgery.. Trans Am Ophthalmol Soc.

[4] Varma D, Adams WE, Phelan PS, Fraser SG (2006). Viscogonioplasty in patients with chronic narrow angle glaucoma.. Br J Ophthalmol.

[5] Chylack LT, Leske MC, McCarthy D, Khu P, Kashiwagi T, Sperduto R (1989). Lens opacities classification system II (LOCS II).. Arch Ophthalmol.

[6] Foster PJ, Aung T, Nolan WP, Machin D, Baasanhu J, Khaw PT, Alsbirk PH, Lee PS, Seah SK, Johnson GJ (2004). Defining “occludable” angles in population surveys: drainage angle width, peripheral anterior synechiae, and glaucomatous optic neuropathy in east Asian people.. Br J Ophthalmol.

[7] Foster PJ, Buhrmann R, Quigley HA, Johnson GJ (2002). The definition and classification of glaucoma in prevalence surveys.. Br J Ophthalmol.

[8] Landers J, Martin K, Sarkies N, Bourne R, Watson P (2012). A Twenty-Year Follow-up Study of Trabeculectomy: Risk Factors and Outcomes.. Ophthalmology.

[9] Kiuchi Y, Tsujino C, Nakamura T, Otori Y, Mochizuki H (2010). Phacoemulsification and trabeculotomy combined with goniosynechialysis for uncontrollable chronic angle-closure glaucoma.. Ophthalmic Surg Lasers Imaging.

[10] Fang AW, Yang XJ, Nie L, Qu J (2010). Endoscopically controlled goniosynechialysis in managing synechial angle-closure glaucoma.. J Glaucoma.

[11] Lai JS, Tham CC, Lam DS (2002). Incisional surgery for angle closure glaucoma.. Semin Ophthalmol.

[12] Feng ZX, Sun NX, Zhang XH, Ren BC (2007). Phacoemulsification or combined with Goniosynechialysis in the management of primary angle-closure glaucoma.. International Journal of Ophthalmology.

[13] Li CX, Tao J, Ji JB, Xiao WW (2008). Long-term observation of phacoemulsification with posterior chamber intraocular lens Implantation and goniosynechialysis in the management of acute angle-closure glaucoma.. Clinical Journal of Ophthalmology.

[14] Zeng LZ, Liu H (2009). Changes of chamber angle after phacoemulsification combined with goniosynechialysis for primary angle-closure glaucoma.. Acta Academiae Medicinae Militaris Tertiae.

[15] See JL, Aquino MC, Aduan J, Chew PT (2011). Management of angle closure glaucoma.. Indian J Ophthalmol.

